# 9. DOES BIOLOGY READ THE DSM? TRANSDIAGNOSTIC FINDINGS IN PSYCHOSIS AND IMPLICATIONS FOR TREATMENT

**DOI:** 10.1093/schbul/sby014.028

**Published:** 2018-04-01

**Authors:** Michael Owen

**Affiliations:** Cardiff University

## Abstract

**Overall Abstract:** A major emerging issue in schizophrenia research is the degree to which the mechanisms underlying the disorder are specific to schizophrenia or are common to a number of disorders, potentially indicating common and distinct pathways to illness. Understanding this is important for diagnosis, biomarkers and the development of new treatments. This symposium will bring together new data to consider the latest findings from different genetic, imaging and clinical approaches.

Dr. Owen, Wales, will present the latest genetic data from the largest genome-wide genetic analyses to date in psychotic disorders (schizophrenia and bipolar disorder) and neurodevelopmental disorders (autism, intellectual disability and ADHD), comprising samples from over 100,000 patients and controls. These data identify novel shared pathways involving neurodevelopmental genes, synaptic function and histone modification that are common across these disorders, but also identify differences in the degree of involvement of particular pathways.

Dr. Howes, England, will present new data from neurochemical and structural imaging studies comparing patients across psychotic and neurodevelopmental disorders (including schizophrenia, bipolar disorder people at risk of autism), showing that there are common dopaminergic alterations linked to psychosis across disorders, as well as showing that structural and neurochemical brain heterogeneity is increased in most brain regions, but also identifying key cortical and sub-cortical regions with increased homogeneity.

Dr. Clementz, USA, will present new EEG and cognitive data from over 400 patients with schizophrenia, and bipolar disorder. This shows differences in intrinsic neural activity that cuts across diagnoses, identifying sub-types that were linked to differences in cognitive functioning.

Dr. Wichers, Netherlands, will present new data from a longitudinal study of adolescents using in-depth real-time phenotyping using experience sampling to investigate the relationship between the coherence of responses and the subsequent development of psychotic and other symptom domains one year later. Her novel application of complex systems theory identifies suspiciousness as a common predictor of the later development of a number of symptoms, but also that other responses, such as low mood, determine the specificity of later outcomes to psychosis.

Overall this symposium will bring together researchers using different, complementary approaches to provide a comprehensive and multi-disciplinary analysis. By bringing researchers from different disciplines together it will enable common mechanisms to be considered, and new insights to be developed. Finally, Dr. DeLisi’s wide-reaching experience means she is well placed to lead the discussion of the implications of these findings for understanding the neurobiology of schizophrenia, and for biomarker development as well as considering their implications for the developing new treatments.

The symposium includes gender diversity in presenters and chairs, speakers from multiple institutions across continents, and diversity in career levels with speakers from early, mid and established positions.

